# Reliability of the 44-question Home Fall Hazard Assessment Tool and personal characteristics associated with home hazards among the Thai elderly

**DOI:** 10.12688/f1000research.126690.1

**Published:** 2023-01-04

**Authors:** Yuwadee Wittayapun, Jiraphat Nawarat, Sarawut Lapmanee, Lynette Mackenzie, Charupa Lektip

**Affiliations:** 1 Department of Physical Therapy, School of Allied Health Sciences, Walailak University, Nakhon Si Thammarat, 8016, Thailand; 2 Movement Science and Exercise Research Center, Walailak University, Nakhon Si Thammarat, 80160, Thailand; 3 Department of Basic Medical Sciences, Faculty of Medicine, Siam University, Bangkok, 10160, Thailand; 4 Discipline of Occupational Therapy, School of Health Sciences, Faculty of Medicine and Health, The University of Sydney, Sydney, New South Wales, 2141, Australia

**Keywords:** elderly, falling, hazard control, prevention, reliability

## Abstract

**Background: **The 44-question Thai Home Fall Hazard Assessment Tool (Thai-HFHAT) was developed to assist healthcare professionals in identifying the risk of falls among community-dwelling elderly from their home environment. However, the reliability of this tool has not been studied. This study aimed to examine reliability of the 44-question Thai-HFHAT and determine the person characteristics associated with home hazards.

**Methods: **A descriptive cross-sectional study design was used for this research. The participants in this study were 51 elderly people from various types of Thai houses: a one-story elevated house, a one-story non-elevated house, and a house with two or more floors, 51 caregivers of elderly patients and 5 village health volunteers (VHV). All participants answered 44 Thai-HFHAT questions to determine inter-rater and test-retest reliabilities. The reliabilities were analyzed using an intra-class correlation coefficient (ICC). Personal characteristics including sex, occupation, and education were used to identify the factors affecting home hazard and linear regression was used to analyze.

**Results: **The ICC of inter-rater reliability of the 44-question Thai-HFHAT was 0.74 (95% CI: 0.57-0.84) and the test-retest reliability was 0.80 (95% CI: 0.64-0.88) for the elderly, 0.80 (95% CI: 0.65-0.89) for the caregivers and 0.70 (95% CI: 0.477-0.83) for the VHV. Personal business career and education level grade 1-3 are less than 0.05, which showed these factors had a significant relationship with the 44-question Thai-HFHAT score as dependent variable.

**Conclusions: **The 44-question Thai-HFHAT is suitable for the home hazards assessment among the elderly in Thailand. Further studies are needed to investigate changes in the house environment after using the 44-question Thai-HFHAT to determine which changes can reduce the risk of fall.

## Introduction

Falls among the elderly are considered a major public health problem, becoming the second leading cause of death and unintentional injuries ^1^ ( https://www.who.int/news-room/fact-sheets/detail/falls). Thailand’s Department of Disease Control has predicted that during 2017-2021, falls among the Thai elderly will account for 27.0% of deaths in the elderly, resulting in a death rate due to falls among Thais of 50 per 100,000 populations https://www.dop.go.th/th/know/side/1/1/1159.

The precipitating cause for falls in the elderly involved the interaction of various risk factors categorized as intrinsic or extrinsic. ^2^ Muscle weakness of the lower extremities and balance impairment were the most important intrinsic factors for the fall. ^3^ In addition, extrinsic factors such as poor housing conditions, inadequate lighting, or slippery floors were also considered mediators in precipitating falls. ^4^ However, most of the evidence comes from high-income countries. Despite the significant burden of falls, prevention strategies are not prioritized in the policy agendas of government in low- and middle-income countries. ^5^ Therefore, identifying potential hazards in Thai houses with an appropriate home hazard screening tool is an effective measure to prevent falls and reduce the risk of falls among the elderly. ^6^

In Thailand, Thai Fall Risk Assessment Test (Thai-FRAT) is a widely used tool to screen risks of fall. ^7^^–^^9^ Of a total of 6 items of Thai-FRAT, there is only one item to evaluate home environmental risk: “Do you live in a traditional Thai house built with an elevated ground floor exceeding 1.5 meters?”. Therefore, the 69-question Thai Home Falls Hazards Assessment Tool (Thai-HFHAT) was designed as a self-reported screening tool to assess the risk of falls at home and is considered suitable for use in Thailand. Psychometric properties of the 69-question Thai-HFHAT were acceptable. ^10^ However, it is time-consuming and difficult for elderly users to precisely complete all questions.

A subsequent study investigated the development of the 44-question Thai-HFHAT based on the instrument design and methodology of the original Thai-HFHAT. The Cox proportional hazard model using stepwise variable selection methods was used to re-design the 69-question Thai-HFHAT. ^11^ There was a report of psychometric properties of 44-question Thai-HFHAT as the adjusted hazards ratio (HR) was 1.26 (95% CI: 1.20-1.33), a cut-off was 18 points, the sensitivity and specificity were 0.93 and 0.72, and the area under the receiver operating characteristic curve (AuROC) was 0.90. ^12^ In addition, the study also found that the 44-question Thai-HFHAT requires only 30 minutes for elderly users to complete all the questions. Occasionally, due to the inability to answer all the elders' questions in practice, the remaining questions were answered by a caregiver or a village health volunteer for the elders. However, the reliability of this tool has not been studied. In order for the 44-question Thai-HFHAT to have psychometric properties in all aspects, a reliability study is required.

Studies on home hazards frequently investigate the area where the hazards are present, the numbers of hazards in the home and how these hazards could contribute to falls. ^13^^,^^14^ Such a unilateral approach does not take into account the characteristics of elderly and how these might make the home environment more hazardous. Romli *et al.* was to investigate the elderly’s characteristics that contribute to home hazards. Lower educational attainment, greater number of home occupants, lower monthly expenditure, and younger age were the factors associated with home hazards. ^15^ The researcher proposes that elderly Thai participants’ characteristic will correlate with the number of home hazards Therefore, this study also aimed to determine the Thai elderly’s characteristic factors contributing to home hazards.

## Methods

### Ethical approval statement

This study was approved by Institutional Review Board of Walailak University (approval number WUEC-20-302-01) on September 16, 2020.

### Informed consent

Written informed consent was obtained from each of the participants.

### Study design

A descriptive cross-sectional study design was used to study the area at risk of falling, personal factors, and reliability of the 44-question Thai-HFHAT. This study was conducted in Tha Khuen Sub-District, Tha Sala District, Nakhon Si Thammarat.

### Participants

The target population consisted of Thai elderly aged 60 years or over, with a total number of 2,552 adults residing in Tha Khun Subdistrict, Tha Sala District, Nakhon Si Thammarat Province ( https://www.dop.go.th/th/know/1). Inclusion criteria were those who achieved fluency in the Thai language. Exclusion criteria were those who could not perform activities of daily living (ADLs) according to Barthel ADLs Index and had dementia determined by the Mini Mental State Examination-Thai 2002. ^16^ Researchers approached the particicpants at their homes to explain the study. 51 elderly people, who passed the exclusion and exclusion criteria as above, were chosen because this number was adequate for examining inter-rater reliability and test-retest reliability. ^17^ Subjects were selected by stratified and quota sampling and categorized according to three types of Thai houses: one-story elevated house, one-story non-elevated house, and a two or more-story house. In addition, 51 caregivers who spend the most time caring for the elderly and five village health volunteers (VHV) with more than five years of experience were also recruited to examine inter-rater reliability for the level of reliability of the 44-question Thai-HFHAT. These different groups were chosen to help identify whether each group of subjects can be replaced by other groups when assessing the hazards in the event the elderly subject cannot complete the instrument by themselves in real life.

### Research instruments

**The 44-question Thai-HFHAT**

The Thai-HFHAT is composed of 44 questions grouped into 7 sections/rooms. 4 items were used to assess hazards in a living room, 4 in a kitchen room, 5 in a garage, 6 for house curtilage, 7 in stairs, 8 in a bedroom, and 10 in a bathroom. Also, the instrument contained a drawing for each room to help the elderly to identify hazards more easily.

**The Barthel Activities of Daily Living Index**

This is an assessment tool for evaluating ADLs for the performance of daily activities by elderly in 10 activities. ^16^ The elderly participants were then classified into three groups according to the scores received: those who were completely independent and able to help others (ADL scores: ≥12), those who were moderately dependent and spent most of their time in their home (ADL scores: 5-11), and those who were completely dependent or disabled (ADL scores: 0-4).

**The Mini Mental State Examination-Thai 2002 (MMSE-Thai 2002)**

This is a Thai version of the cognitive impairment assessment tool for the Thai elderly. ^16^ The cognitive impairment of the elderly can be preliminarily determined when the elderly who received no formal education had MMSE-Thai scores of ≤14, when the elderly who received only upper secondary education had scores of ≤17, and when the elderly who continued their education received a score of ≤22.

### Data collection

Data on demographic characteristics of the elderly, caregivers, and VHVs were collected. Three groups of study subjects were asked to fill out the 44-question Thai-HFHAT. They were instructed to enter each room in their home and answer a list of questions for assessing fall hazards in each room using a guided drawing. Scoring of potential hazards from the screening tool was performed. High scores have been associated with an increased risk of falls. The study subjects were informed to complete the screening tool within 30 minutes. The hazard areas, the subject’s characteristic factors, and inter-rater reliability were conducted after obtaining data from all subjects.

All three groups were instructed to perform the second assessment a week later ^15^ so that researchers could collect more data for examining test-retest reliability. During the assessment, the elderly, caregivers, and VHV subjects had to independently answer assessment questions, and no conversation was permitted. We considered the intraclass correlation coefficient (ICC) with values ranging from 0 to 1 suitable for the evaluation of inter-rater and test-retest reliability. The ICC results were classified as follows: values between 0.00-0.49 were classified as poor reliability, values between 0.50-0.74 as moderate, values between 0.75-0.90 as good, and 0.91-1.00 as excellent. ^18^ Data collection started in August 2020 and ended in September 2020.

### Data analysis

All data were recorded and entered using the statistical package software version 22 (SPSS Inc. Chicago, IL, USA). Mean and standard deviation (SD) were used to analyze the subjects’ characteristics. Frequency and percentage were used show the data of home hazard areas. Inter-rater and test-retest reliabilities were evaluated using an intra-class correlation coefficient (ICC), that is, ICC (2, k) and ICC (3, k), respectively. Mean score differences between the elderly, caregivers, and VHV subjects were evaluated using a One-Way ANOVA. Differences in mean scores from the first and the second visits (1 week apart) were analyzed using an independent-samples t-test. We used multiple linear regression to predict the independent factors, consisting of sex, occupation, and education level, affecting the 44-question Thai-HFHAT score. ^19^^,^^20^ Information bias may arise from the use of the 44-question Thai-HFHAT. The researcher explained the assessment tool to the participants to help them understand the objectives, and that the presentation of the results that will be anonymized. So, nobody knows how each subject will be evaluated, so that subjects are confident that there will be no impact from the actual assessment.

## Results

This study included 107 study subjects, and the demographic characteristics of all subjects and fall history of the elderly subjects are shown in Tables 1 and 2, respectively. 59% of the elderly lived in a one-story non-elevated house, 55.8% had blurred vision, and 86.5% demonstrated normal balance ability (≥10 seconds of tandem standing). The underlying diseases of the elderly subjects included hypertension (46.2%) and hyperlipidemia (44.2%). Most caregivers had a close relationship with the elderly subjects (53.9%). The mean (±SD) duration of caregiving in the caregiver group was 21.73 (±5.71) hours/day or 6.88 (±0.83) days/week. The mean (±SD) working experience of the VHV subjects was 12.96 (±6.63) years.

**Table 1.** Demographic characteristics of study subjects.

Characteristics	Elderly (n = 51)	Caregiver (n = 51)	VHV (n = 5)	*p*-value
	n (%)	n (%)	n (%)	
**Sex**				0.143
Men	20 (39.2)	23 (45.1)	0 (0.0)	
Women	31 (60.8)	28 (54.9)	5 (100.0)	
Mean ± SD age in years	73.40 (7.21)	58.12 (15.82)	45.5 (6.24)	< 0.001*
Mean ± SD BMI in kg/m ^2^	25.67 (1.23)	22.71 (3.81)	22.33 (4.45)	0.182
**Education level**				< 0.001*
Grades 1-3	44 (86.3)	19 (37.3)	1 (20.0)	
Grades 4-6	3 (5.9)	12 (23.5)	3 (60.0)	
Above grade 6	4 (7.8)	20 (39.2)	1 (20.0)	
**Marital status**				0.913
No partner	6 (11.8)	6 (11.8)	1 (20.0)	
Have a partner	45 (88.2)	45 (88.2)	4 (80.0)	
**Occupations**				0.086
Housekeeper	30 (58.8)	19 (37.3)	2 (40.0)	
Agriculture	12 (23.5)	23 (45.1)	2 (40.0)	
Personal business	9 (17.7)	9 (17.7)	1 (20.0)	

VHV, village health volunteers.

**Table 2.** Fall history of the elderly subjects (n = 51).

Falling history	n (%)
**History of falling in the previous 6 months**	
Yes	14 (27.5)
No	37 (72.6)
**The cause of falling**	
Tripping	8 (57.1)
Loss of balance	5 (35.7)
Slip	1 (7.1)
**Fall injury**	
No	7 (13.5)
Sprain	3 (5.8)
Scratch	3 (5.8)
Fracture	3 (5.8)

**Figure 1.**
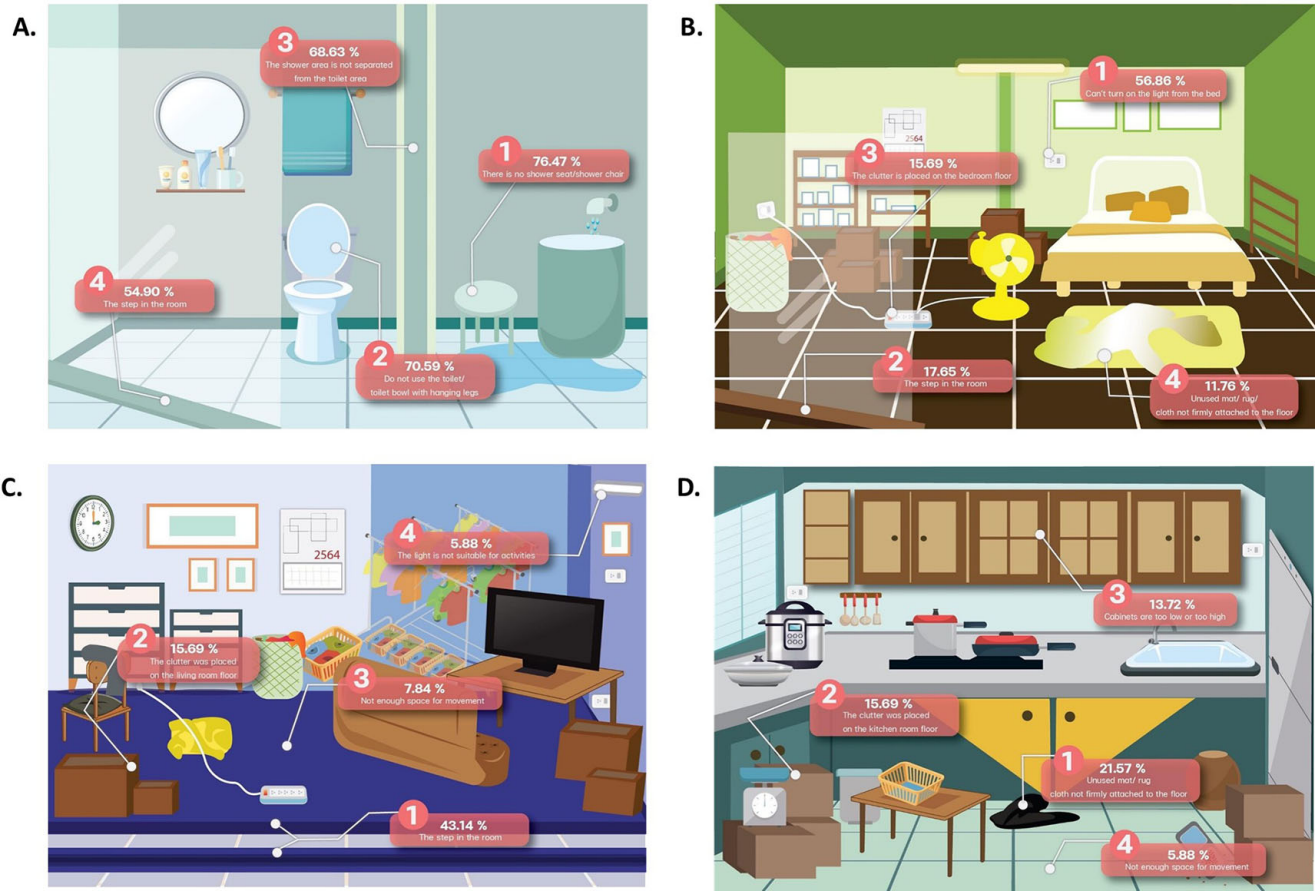
The areas at risk of fall in home.

The areas at the most risk of fall risk are the bathroom (94.1%), bedroom (74.5%), living room (56.9%), kitchen room (37.3%), around the home (35.3%), garage (25.5%), and stair in the home (7.8%), respectively. The risk of falling in each room is shown in Figure 1.

### Inter-rater reliability

**Figure 2.**
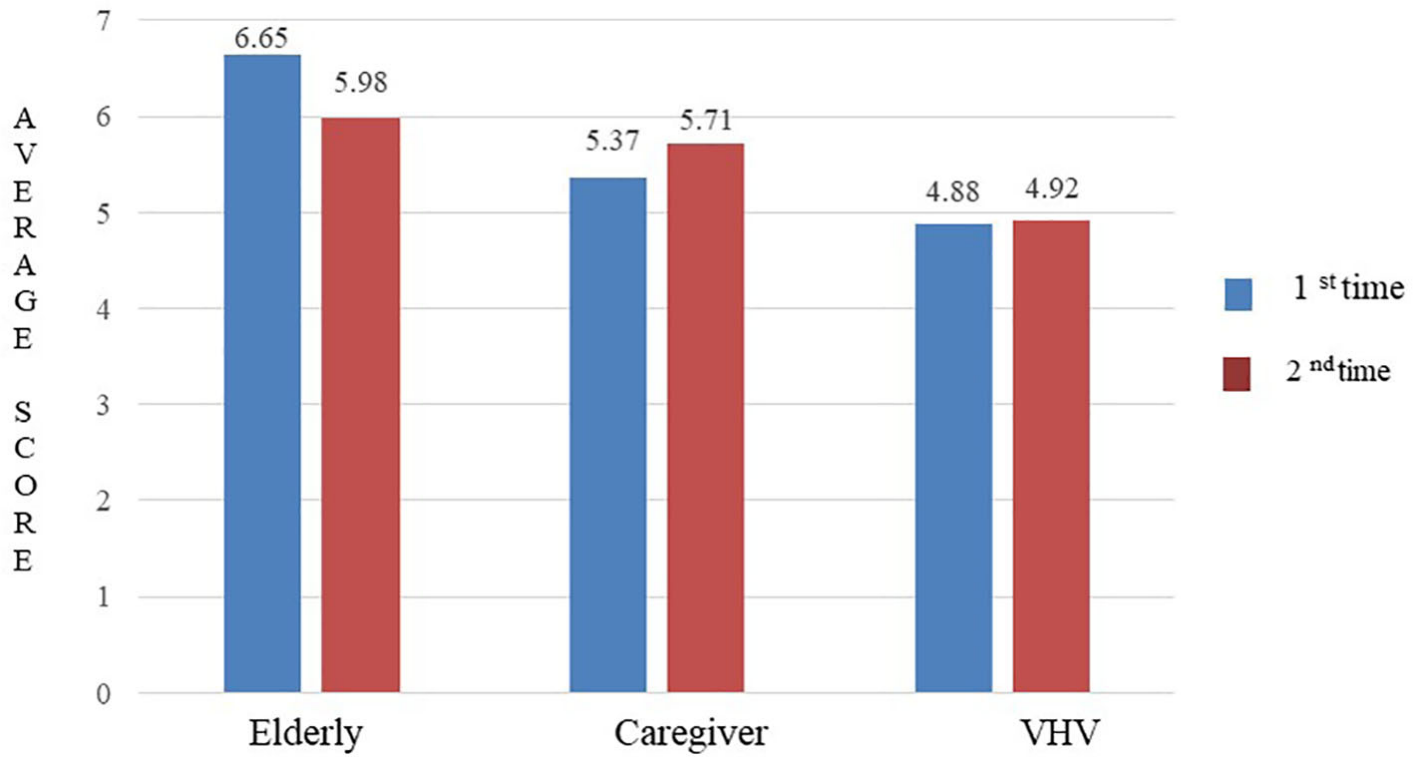
The average scores of Thai-HFHAT for the 1 ^st^ and 2 ^nd^ time of the subjects.

The ICC for the 44-question Thai-HFHAT was 0.74 (95% CI: 0.57-0.84). The mean (±SD) scores of the elderly, caregiver, and VHV groups were 6.65 (±3.29), 5.37 (±3.22), and 4.88 (±2.65), respectively. The mean difference in scores for all three groups was statistically significant ( *p* = 0.012), as shown in Figure 2.

### Test-retest reliability

The ICC for the 44-question Thai-HFHAT was 0.80 (95% CI: 0.64-0.88) for the elderly group, 0.80 (95% CI: 0.65-0.89) for the caregiver group, and 0.70 (95% CI: 0.48-0.83) for the VHV group. The mean difference in scores obtained before and after one week of the assessment of the elderly ( *p* = 0.283), caregiver ( *p* = 0.604), and VHV ( *p* = 0.984) groups was not statistically significant. The average scores of the 1 ^st^ and 2 ^nd^ time of the elderly, caregivers, and VHV are shown in Figure 2.

### Personal characteristics associated with home hazards

As shown in Table 3, the values of variance inflation factor (VIF) are all less than 5 and all the tolerance values are more than 0.10. ^19^ The *p*-value for all independent variables, that is, personal business career and education level grade 1-3 are less than 0.05, which shows they have a significant relationship with the dependent variable, 44-question Thai-HFHAT score.

**Table 3.** Regression analysis for predicting 44-question Thai-HFHAT score ^a^.

Variable	Unstandardized coefficients		Standardized coefficients			Collinearity statistics	
	B	SE	Beta	T	Sig.	Tolerance	VIF
(Constant)	3.13	1.72		1.82	0.071		
Sex	-0.34	0.64	-0.05	-0.54	0.593	0.82	1.22
Agriculture	-0.12	0.73	-0.02	-0.17	0.865	0.68	1.48
Personal business	-2.05	0.95	-0.23	-2.15	0.034	0.89	1.12
Education grade 1-3	3.99	1.64	0.59	2.43	0.017	0.51	1.96
Education grade 4-6	2.43	1.66	0.35	1.47	0.146	0.55	1.81

SE: Standard Error; VIF: Variance Inflation Factor.

^a^ Dependent variable: 44-question Thai-HFHAT score (r = 0.41, r ^2^ = 0.17, Adj. r ^2^ = 0.12).

## Discussion

37 elderly participants in this study (72.6%) had no history of falls. This may be attributed to regular exercise of the subjects, as the 34 subjects (66.6%) performed a regular exercise routine. Our results are consistent with the study by Hopewell S. *et al.* (2018) who reported that the practice of regular exercise would decrease fall rates and reduce the risk of falls in the elderly. ^21^ Of all elderly surveyed subjects, 27.5% reported a fall. This number is close to that predicted by the Thailand Department of Disease Control report on the prevalence of falls during 2017-2021, in which falls among Thai elderly account for 27.0% ( https://www.dop.go.th/th/know/side/1/1/1159). The area of the home with the most falls was the bathroom at 94.1 %, consistent with several studies on both Thais and in other countries. ^22^^–^^25^ The bathrooms are areas where water is trapped with no separation between wet and dry areas. In addition, the present step in the room, no toilet or seat with hanging legs, and no shower seat/shower chair are causes of most falls in the bathroom.

The inter-rater reliability of the study subjects using the 44-question Thai-HFHAT was moderate (ICC = 0.74) and the test-retest reliability among the elderly was good (ICC = 0.80). Our results indicated that the 44-question Thai-HFHAT is as reliable as the 69-question Thai-HFHAT, whose inter-rater reliability was good (ICC = 0.87) and the test-retest reliability was good (ICC = 0.87). In our study, the 44-question Thai-HFHAT had lower ICC than the 69-question Thai-HFHAT. This is probably because the study was conducted with 30 elderly and caregiver participants and 1 VHV participant, resulting in the moderate level of inter-rater reliability. ^10^ The smaller sample size of the 69-question Thai-HFHAT slightly affected the evaluation of reliability, causing minor errors. However, the inter-rater reliability of the 44-question version was similar to that of the 69-question version.

The inter-rater reliability of the 44-question version was higher than the Modified HOME FAST-SR (Thai version) (ICC = 0.64). ^12^ This is probably because the Thai-HFHAT was designed to have questions listed in an organized manner with drawings to help illustrate each room in a house, allowing subjects to identify home hazards at ease. However, the text in the HOME FAST-SR may have been confusing. For instance, in the HOME FAST-SR question 8b asks “Does it take you several attempts to get up out from your sitting chair?”, and question 8c asks “When you lower yourself into the chair, can you do it without falling back on the chair?”. These two statements may have caused confusion that could lead to medical measurement errors. ^26^

We found that the elderly subjects had a higher mean home hazard rating, followed by caregivers and VHV subjects. This is likely because most of our elderly subjects had a health issue and considered falling one of the health issues that cause the most damage, ^27^ prompting the elderly to pay more attention to risk factors that contribute to falls than caregiver and VHV groups. Also, the difference in mean home hazard ratings between the elderly and VHV subjects was statistically significant ( *p* = 0.012). Our results are consistent with the previous study by Morgan *et al.* (2005) who investigated the reliability of a self-report home hazard screening tool and found some questions, i.e. “Is lighting suitable for activities?”, could not be precisely answered by looking around the home environment. Such questions were viewed by the elderly subjects as increasing the risk of falls, whereas the VHV subjects may not. ^28^ Thus, the self-report 44-question home hazard screening tool was preferred for the home hazards assessment among the elderly. In our study, the mean ratings of the 44-question Thai-HFHAT among the elderly, caregivers, and VHV groups were varied in the first and second assessments. We found a slight decrease in the mean rating of the elderly subjects on the second visit. This may be due to changes in the behavior of the elderly subjects and in the home environment between the first and the second visits. The study subjects may have removed obstacles like power cords from walkways before the second home visit. This phenomenon is called “reactivity” and can occur as a result of administering an instrument to the study subjects multiple times. Subjects become sensitized with the instrument and “learn” to respond when they perceive how they are expected to respond. ^29^

Elderly participants with higher education levels had lower number of home hazards. These elderly participants might have greater awareness and more access information to assist with improving the safety of their home environment. Higher educational attainments are likely to be associated with better income and socioeconomic status, and therefore greater affordability for safer housing and home modification. ^30^ Moreover, home hazards appeared to be associated with occupation. This study found that the group who were working in their own homes as housekeepers had a higher risk of falling, which may be caused by the clutter in the home.

The main limitation of our study was the small number of the sample size. To achieve the valid generalization that covers most types of Thai houses, this study should have been conducted with a larger sample size to ensure the applicability of the screening tool. Further studies are needed to investigate the changes in house environment after using the 44-question Thai-HFHAT to determine what particular changes could reduce fall risk. Finally, the 44-question Thai-HFHAT was developed in the Thai version. Therefore, cross-cultural translation of 44-question Thai-HFHAT is important for widespread use.

## Conclusions

Our study confirmed that the 44-question Thai-HFHAT is suitable for the home hazards assessment among the elderly in Thailand.

## Author contribution

Conceptualization, CL; Data curation, CL, YW; Investigation, CL, YW, JN, SL, LM; Methodology, CL, YW, JN, LM; Project administration, CL; Supervision, JN, SL, LM; Writing-original draft, CL, SL; Writing-review & editing, CL, RP, JN, SL, LM.

## Data Availability

figshare: Underlying data and extended data of Reliability on the 44-question Home Fall Hazard Assessment Tool and Personal Characteristics Associated with Home Hazards among thein Thai Elderly. https://doi.org/10.6084/m9.figshare.c.6239961.v1 ^31^
‐ General information of elderly. https://doi.org/10.6084/m9.figshare.21382278
‐ General information of caregiver. https://doi.org/10.6084/m9.figshare.21343374
‐ General information of village health volunteer. https://doi.org/10.6084/m9.figshare.21343380
‐ 1st test done by elderly. https://doi.org/10.6084/m9.figshare.21343383
‐ 1st test done by caregiver. https://doi.org/10.6084/m9.figshare.21343413
‐ 1st test dons by VHV. https://doi.org/10.6084/m9.figshare.21343431
‐ 2nd test done by elderly. https://doi.org/10.6084/m9.figshare.21343464
‐ 2nd test done by caregiver. https://doi.org/10.6084/m9.figshare.21343470
‐ 2nd test done by VHV. https://doi.org/10.6084/m9.figshare.21343476 - Supplementary Table 1. https://doi.org/10.6084/m9.figshare.21304887
- Inform consent. https://doi.org/10.6084/m9.figshare.21304920 Data are available under the terms of the Creative Commons Attribution 4.0 International license (CC-BY 4.0).
