# Supplementary material for: Reliability of the 44-question Home Fall Hazard Assessment Tool and demographic characteristics associated with home hazards among the Thai elderly
Source: F1000Res. 2023 Jul 19;12:8. Originally published 2023 Jan 4. [Version 4] doi: 10.12688/f1000research.126690.4 (PMC10336358; doi:10.12688/f1000research.126690.4)
Supplement: Peer review history

# Peer review: Reviewer response for version 3

**DOI:** 10.5256/f1000research.148757.r183646

**Published:** 2023-07-11

**Authors:** Machiko R Tomita

**Affiliations:** 1 Department of Rehabilitations Science, University at Buffalo, Buffalo, NY, USA

The revised manuscript reads well. I noticed the lack of mentioning about statistical analysis software used (SPSS) in this manuscript. It is a good practice to reveal it.

Is the work clearly and accurately presented and does it cite the current literature?

Partly

If applicable, is the statistical analysis and its interpretation appropriate?

Partly

Are all the source data underlying the results available to ensure full reproducibility?

Partly

Is the study design appropriate and is the work technically sound?

Partly

Are the conclusions drawn adequately supported by the results?

Yes

Are sufficient details of methods and analysis provided to allow replication by others?

Partly

Reviewer Expertise:

Fall prevention, smart house, e-health in gerontological population.

I confirm that I have read this submission and believe that I have an appropriate level of expertise to confirm that it is of an acceptable scientific standard.

# Response

**Published:** 2023-07-13

**Authors:** Charupa Lektip

**Affiliations:** Walailak University, Thailand

1) Changed the title from "Reliability of the 44-question Home Fall Hazard Assessment Tool and **personal characteristics** associated with home hazards among the Thai older adults" to "Reliability of the 44-question Home Fall Hazard Assessment Tool and **demographic characteristics** associated with home hazards among the Thai older adults.

 2) Mention the statistical analysis software used (SPSS) as "Data were analyzed using the statistical package for the social sciences (SPSS), version 22.0 for Windows (IBM Corporation, New York, NY, USA)"

# Peer review: Reviewer response for version 2

**DOI:** 10.5256/f1000research.144999.r165889

**Published:** 2023-05-18

**Authors:** Machiko R Tomita

**Affiliations:** 1 Department of Rehabilitations Science, University at Buffalo, Buffalo, NY, USA

This article has been improved, but being viewed with fresh eyes, there are some issues that need to be addressed by authors.

 Major issue: What was the reason that fall related personal characteristics were not included in the study using regression? Age, past falls, past fall injuries, physical and cognitive function, balance/dizziness, foot problems, alcohol intake, incontinence, vision and hearing problems, high bald pressure, etc. It looks like you have information about some of them. If you are interested in demographic factors only, you should have controlled for these fall risk factors that are known to cause a fall. To do so, you need more than 51 participants to use a regression analysis.

 Minor issues: These are mainly for writing clarifications and are easy to fix.

**Abstract**

 1. In Background, use the word “demographic” rather than “personal” characteristic because in Methods, only sex, occupation, and education are mentioned. Why was age not included?

 2. Choose either “interrater” or “inter-rater”.

 3. In Results, the p-value should be less than .05. If it is significantly related, it should be p≤.05; therefore, it is not necessary to talk about it. Revise the sentence to something such as “In demographic variables, business career and education level (grades 1-3) had significant relations with the total number of home hazards in the 44-questions Thai-HFHAT.”

**Introduction**

 4. Words such as “the elderly” and “elderly” are not preferred terms. Use “older adults”, instead. This is for the whole manuscript.

 5. Can you be a little more specific about poor housing conditions?

 6. How many minutes does it take to complete the 69-question Thai HFHAT while 44-quesitons take 30 minutes?

**Methods**

 7. In Study design, a description is only for interrater reliability. Write one for test-retest reliability. They are written in the Abstract.

 8. Start a new sentence for determination of dementia using MMSE-Thai 2002 or follow my suggestion for # 12 below.

 9. 51 should be spelled out. Same for 4 items in Research and 37, the beginning in Discussion when a sentence starts with a number.

 10. The word “passed” should be replaced with “met”.

 11. After “this number was adequate” it is better to say, “for our primary purpose – examining interrater and test-retest reliability.” It is because if you used only 51 participants for regression, it is not enough. The parts talking about the sample sizes are a bit confusing. Please clarify the sample size for each of three analyses. You had these 3 groups for interrater reliability study, which was one of the study strengths.

 12. The information about MMSE-Thai 2002 is redundant with that in Participants. This part is easier to understand than in Participants section. It may be better to leave it something like “ had dementia determined by the Mini Mental State Examination-Thai 2002. Do you have the information about psychometric properties of MMSE-Thai 2002?

 13. In Data collection, if participants were instructed to complete the assessment within 30 minutes, you have to revise the sentence In the Introduction, “requires only 30 minutes for elderly users to complete”. It is because we read the sentence that “older participants took 30 minutes to complete” and you are reporting the fact. The revised sentence should be “…was designed for older participants to complete it within 30 minutes.”

 14. Were 51 older participants’ houses used for reliability tests?

 15. Provide one or two examples of instructions or questions to identify a home hazard. Were they asked, “Isthis room lighted well?” “Does thisroom light well not to fall?”, etc.?

 16. “So that reserachers could collect more data for examining test-rest reliability,” should be “for test-retest reliability.”

 17. In Data analysis, citation (SPSS Inc., Chicago, IL, USA) should be replaced with (IBM Corp. Armonk, NY, USA). IBM bought SPSS in 2009.

 18. Was the test-retest performed for the three groups separately or altogether? Later I understood but it should be written here also.

 19. Sentences starting with “Information bias” are not necessary. It should be covered by IRB. If you must write it for some reason, it should be placed in the Data collection section.

**Results**

 20. Caregivers provided care about 22 hours/day on average. The hour seems extremely high even if care recipients have dementia.

 21. In Table 2, 14 participants fell but in fall injury, 16 participants answered. The total should be 14. Also, the percentage should be calculated out of 14.

 22. The result section should be written in past tense.

 23. Is the sentence starting with “the area at the most risk of fall risk are…” 94.1% of 107? Revise the sentence, such as “The most home hazards that may cause a fall was found for the bathroom (94.1% of 107), then bedroom…” Did 94.1% of participants identify at least one of 4 items in the bathroom is a fall risk?

 24. “The risk of falling in each room” and the title of Figure 1 should be replaced by for example “Percentage of identified home hazards that may cause a fall in each room.”

 25. Who are participants for personal characteristics associated with home hazards? 51 older adults?

 26. Why was age not included in independent variables? Please read a major concern.

 27. In Table 3, all r should be R. Discussion 28. I would use (91.9% of 37 non-fallers) rather than (66.6%).

 29. I would state “This figure supports the study by Hopewell….” rather than “Our results are consistent..” because this was not the purpose of the study and happened to find it.

 30. Remove the last sentence of the first paragraph on page 8, staring “This makes it more reliable than….”. When raters are homogeneous, they tend to rate in a similar manner. In your study, the raters are diverse. Accordingly, the reference 26 should move after (ICC=0.74)” When we use 1 vs. K, the use of ICC(2,k) produces a larger ICC value than ICC (2,1). It is not always that a lager sample size produces a lower ICC value than a small sample size. When there is little variation among raters given to each item, then the ICC v values become high. Revision of these paragraphs related to the sample size and ICC values is recommended or if the statement is true, put citations.

 31. You discussed a lot about interrater reliability but not much about test-retest reliability.

 32. Finally, if you do have answers for my major concern, please state them and if not, you should state lack of control for known fall risks in the limitation.

Is the work clearly and accurately presented and does it cite the current literature?

Partly

If applicable, is the statistical analysis and its interpretation appropriate?

Partly

Are all the source data underlying the results available to ensure full reproducibility?

Partly

Is the study design appropriate and is the work technically sound?

Partly

Are the conclusions drawn adequately supported by the results?

Yes

Are sufficient details of methods and analysis provided to allow replication by others?

Partly

Reviewer Expertise:

Fall prevention, smart house, e-health in gerontological population.

I confirm that I have read this submission and believe that I have an appropriate level of expertise to confirm that it is of an acceptable scientific standard, however I have significant reservations, as outlined above.

# Response

**Published:** 2023-05-23

**Authors:** Charupa Lektip

**Affiliations:** Walailak University, Thailand

**Major issue:** What was the reason that fall related personal characteristics were not included in the study using regression? Age, past falls, past fall injuries, physical and cognitive function, balance/dizziness, foot problems, alcohol intake, incontinence, vision and hearing problems, high bald pressure, etc. It looks like you have information about some of them. If you are interested in demographic factors only, you should have controlled for these fall risk factors that are known to cause a fall. To do so, you need more than 51 participants to use a regression analysis.

**Response**

          The main objective of this study was to study home hazards associated with falls using a developed assessment form. Home hazards were classified as extrinsic factors affecting falls that were also important to reduce the risk of falls from home hazard. Romli et al., 2018 reported that older adult characteristics consist of education, number of home occupants, monthly expenditure, traditional housing, ethnicity, and age were correlated with home hazard, but were not reported for the Thai older adult.

          Therefore, in this study, which was conducted in a Thai population, so interested in the educations, sex, and income-generating occupation, but there is also a limitation on the age variable since the study was conducted in an elderly population that 75 % was middle elderly (70-79 years) and 15% was the late elderly (80-89 years) of the Thai population tended to be low survivors. This is a limitation in the analysis of regression data. The researchers will write a limitation in the next study for clarity.

           The other variables recommended by the reviewer are good recommendations as they are intrinsic factors that affect falls as well. But with the main objective of focusing on home hazards, the researchers did not collect data on these variables. However, inclusion criteria were used by the investigators to attempt to control for intrinsic factors as much as possible. For this reason, I would like to adjust the title to only demographic characteristics and would like to take the reviewer's advice as a limitation and suggestion next time.

**Minor issues:** These are mainly for writing clarifications and are easy to fix.

**Abstract**

 1. In Background, use the word “demographic” rather than “personal” characteristic because in Methods, only sex, occupation, and education are mentioned. Why was age not included?

**Response**

 I have changed personal to demographic characteristic.

 Because the study was conducted in an elderly population that 75 % was middle elderly (70-79 years) and 15% was the late elderly (80-89 years) of the Thai population tended to be low survivors. The researchers assumed that the irrelevant age of the subjects would not affect the home hazard. 

 2. Choose either “interrater” or “inter-rater”.

**Response** I have changed interrater to inter-rater.

 3. In Results, the p-value should be less than .05. If it is significantly related, it should be p≤.05; therefore, it is not necessary to talk about it. Revise the sentence to something such as “In demographic variables, business career and education level (grades 1-3) had significant relations with the total number of home hazards in the 44-questions Thai-HFHAT.”

**Response** I have fixed as you suggest.

**Introduction**

 4.    Words such as “the elderly” and “elderly” are not preferred terms. Use “older adults”, instead. This is for the whole manuscript.

**Response** I have change elderly to older adults as you suggest.

 5. Can you be a little more specific about poor housing conditions?

**Response** I have described poor housing in the line 12.

 6. How many minutes does it take to complete the 69-question Thai HFHAT while 44-quesitons take 30 minutes?

**Response **45 minutes.

**Methods**

 7. In Study design, a description is only for interrater reliability. Write one for test-retest reliability. They are written in the Abstract.

**Response** I have described about test-retest in the line 67.

 8. Start a new sentence for determination of dementia using MMSE-Thai 2002 or follow my suggestion for # 12 below.

**Response** I follow as you suggest in item 12.

 9. 51 should be spelled out. Same for 4 items in Research and 37, the beginning in Discussion when a sentence starts with a number.

**Response** I have already change as you suggest in line 82, 98 and 202.

 10. The word “passed” should be replaced with “met”.

**Response** I have already change as you suggest in line 83.

 11. After “this number was adequate” it is better to say, “for our primary purpose – examining interrater and test-retest reliability.” It is because if you used only 51 participants for regression, it is not enough. The parts talking about the sample sizes are a bit confusing. Please clarify the sample size for each of three analyses. You had these 3 groups for interrater reliability study, which was one of the study strengths.

**Response** I already clarify as you suggest about sample size for inter-rater reliability and test-retest reliability as our primary purpose in line 84-89. 

 12. The information about MMSE-Thai 2002 is redundant with that in Participants. This part is easier to understand than in Participants section. It may be better to leave it something like “had dementia determined by the Mini Mental State Examination-Thai 2002. Do you have the information about psychometric properties of MMSE-Thai 2002?

**Response** I have added the information about psychometric properties in the line 116.

 13. In Data collection, if participants were instructed to complete the assessment within 30 minutes, you have to revise the sentence In the Introduction, “requires only 30 minutes for elderly users to complete”. It is because we read the sentence that “older participants took 30 minutes to complete” and you are reporting the fact. The revised sentence should be “…was designed for older participants to complete it within 30 minutes.”

**Response** I have already changed the sentence in the Introduction as you suggest in line 39-40.

     

 14. Were 51 older participants’ houses used for reliability tests?

**Response** Yes, of course.

 15. Provide one or two examples of instructions or questions to identify a home hazard. Were they asked, “Isthis room lighted well?” “Does thisroom light well not to fall?”, etc.?

**Response** I have already added the example of questions in line 126-128.

 16. “So that reserachers could collect more data for examining test-rest reliability,” should be “for test-retest reliability.”

**Response** I have already edited in line 134.

 17. In Data analysis, citation (SPSS Inc., Chicago, IL, USA) should be replaced with (IBM Corp. Armonk, NY, USA). IBM bought SPSS in 2009.

**Response** I have already change as you suggest in line 145.

 18. Was the test-retest performed for the three groups separately or altogether? Later I understood but it should be written here also.

**Response** I have already added “the test-retest performed for the three groups separately” in line 150.

 19. Sentences starting with “Information bias” are not necessary. It should be covered by IRB. If you must write it for some reason, it should be placed in the Data collection section.

**Response** I have already deleted “Information bias” out of data analysis part.

**Results**

 20. Caregivers provided care about 22 hours/day on average. The hour seems extremely high even if care recipients have dementia.

**Response** Most caregivers are family members such as husbands, daughter or son who live in the same house. Each person's care period is almost 24 hours.

 21. In Table 2, 14 participants fell but in fall injury, 16 participants answered. The total should be 14. Also, the percentage should be calculated out of 14.

**Response** I apologize for adding the wrong numbers. I have done the fix as you suggested.

 22. The result section should be written in past tense.

**Response **I have written the result section in the past tense.

 23. Is the sentence starting with “the area at the most risk of fall risk are…” 94.1% of 107? Revise the sentence, such as “The most home hazards that may cause a fall was found for the bathroom (94.1% of 107), then bedroom…” Did 94.1% of participants identify at least one of 4 items in the bathroom is a fall risk?

**Response** I have rewritten the sentence as you suggest. Participants who identify at least one of all questions in each room assume that the fall risk.    

 24. “The risk of falling in each room” and the title of Figure 1 should be replaced by for example “Percentage of identified home hazards that may cause a fall in each room.”

**Response** I have rewritten as suggest in line 179-180 and the title of Figure 1.

 25. Who are participants for personal characteristics associated with home hazards? 51 older adults?

**Response** Yes, the demographic characteristics of 51 older adults. I have added word “older adults” in the topic for easy to understand.

 26. Why was age not included in independent variables? Please read a major concern.

**Response** Because the study was conducted in an elderly population that 75 % was middle elderly (70-79 years) and 15% was the late elderly (80-89 years) of the Thai population tended to be low survivors. The researchers assumed that the irrelevant age of the subjects would not affect the home hazard.

 27. In Table 3, all r should be R. 

**Response** I have replaced r by R as you suggest.

**Discussion**

 28. I would use (91.9% of 37 non-fallers) rather than (66.6%).

**Response** I have changed from 66.6% to 91.9%.

 29. I would state “This figure supports the study by Hopewell….” rather than “Our results are consistent.” because this was not the purpose of the study and happened to find it.

**Response** I have edited as you suggest in line 213-214.

 30. Remove the last sentence of the first paragraph on page 8, staring “This makes it more reliable than….”. When raters are homogeneous, they tend to rate in a similar manner. In your study, the raters are diverse. Accordingly, the reference 26 should move after (ICC=0.74)” When we use 1 vs. K, the use of ICC(2,k) produces a larger ICC value than ICC (2,1). It is not always that a lager sample size produces a lower ICC value than a small sample size. When there is little variation among raters given to each item, then the ICC v values become high. Revision of these paragraphs related to the sample size and ICC values is recommended or if the statement is true, put citations.

**Response** I have moved references 26 to after (ICC=0.74). I've changed the reason for this study's ICC being less than the previous study without justifying the sample size in the line 235-238.

 31. You discussed a lot about interrater reliability but not much about test-retest reliability.

**Response** I added the discussion on test-retest reliability in the same section as the discussion on inter-rater reliability.

 32. Finally, if you do have answers for my major concern, please state them and if not, you should state lack of control for known fall risks in the limitation.

**Response** I have already explained the major concern in the first part of your question. It will be put in the limitation section in line 295-300.

# Peer review: Reviewer response for version 2

**DOI:** 10.5256/f1000research.144999.r165888

**Published:** 2023-03-21

**Authors:** Asmidawati Ashari

**Affiliations:** 1 Department of Human Development and Family Studies, Faculty of Human Ecology, University Putra Malaysia, Serdang, Malaysia

Thank you for your kind responses toward my comments. I am agreed with the revised version.

Is the work clearly and accurately presented and does it cite the current literature?

Yes

If applicable, is the statistical analysis and its interpretation appropriate?

Yes

Are all the source data underlying the results available to ensure full reproducibility?

Partly

Is the study design appropriate and is the work technically sound?

Yes

Are the conclusions drawn adequately supported by the results?

Partly

Are sufficient details of methods and analysis provided to allow replication by others?

Yes

Reviewer Expertise:

Fall prevention and aged care research.

I confirm that I have read this submission and believe that I have an appropriate level of expertise to confirm that it is of an acceptable scientific standard.

# Peer review: Reviewer response for version 1

**DOI:** 10.5256/f1000research.139124.r158960

**Published:** 2023-02-28

**Authors:** Asmidawati Ashari

**Affiliations:** 1 Department of Human Development and Family Studies, Faculty of Human Ecology, University Putra Malaysia, Serdang, Malaysia

The paper by Lektip *et al.* analysed the reliability of the 44-question Thai Home Fall Hazard Assessment Tool (Thai-HFHAT) and determine the person characteristics associated with home hazards. This is an interesting study and the authors have collected a unique dataset using cutting edge methodology.

 Overall, the information presented represents valuable information regarding the feasibility of using (Thai-HFHAT). The main strength of this paper is that it addresses an interesting question and finds a novel solution based on a carefully selected set of rules and provides a clear answer in culturally context. The paper is generally well written and structured. The title and abstract are appropriate for the content of the text.

 However, in my opinion the paper has some shortcomings regarding some data analyses and discussion. The number of subjects for VHV group is too small (5) as compared to other groups. This number is too small which may affect the analysis. I also suggested to add further information on caregiver and VHF personal characteristic to explain any important aspects contributing to the variation in (Thai-HFHAT) score.

Is the work clearly and accurately presented and does it cite the current literature?

Yes

If applicable, is the statistical analysis and its interpretation appropriate?

Yes

Are all the source data underlying the results available to ensure full reproducibility?

Partly

Is the study design appropriate and is the work technically sound?

Yes

Are the conclusions drawn adequately supported by the results?

Partly

Are sufficient details of methods and analysis provided to allow replication by others?

Yes

Reviewer Expertise:

Fall prevention and aged care research.

I confirm that I have read this submission and believe that I have an appropriate level of expertise to confirm that it is of an acceptable scientific standard, however I have significant reservations, as outlined above.

# Response

**Published:** 2023-03-01

**Authors:** Charupa Lektip

**Affiliations:** Walailak University, Thailand

Thanks for the compliment and advice on my research. Allow me to explain the number of 5 VHV, which seems too small for inter-rater and test-retest reliability.

 1) The most important target group used by the 44-question Thai-HFHAT was the elderly, followed by caregivers, while VHV was the final target group in cases where the elderly were unable to use the assessment and lacked caregivers.

 2) In Thailand, one VHV takes care of people in around 10-15 households. Similarly, this study used one VHV to assess 10 elderly homes. In this study, 51 elderly people were studied, so 5 VHVs were used to assess.

# Peer review: Reviewer response for version 1

**DOI:** 10.5256/f1000research.139124.r161483

**Published:** 2023-02-14

**Authors:** Machiko R Tomita

**Affiliations:** 1 Department of Rehabilitations Science, University at Buffalo, Buffalo, NY, USA

The purposes of the study are to establish reliability of 44-item home fall hazard assessment tool for Thai older adults and to find personal characteristics associated with the identified number of fall risks. The reliability section is done correctly but can be improved further.  

1. One of the most comprehensive studies about reliability and validity of home hazard to prevent falls in older adults is missing ^1^. 
2. This study is a methodological study not cross-sectional.
3. A study replication cannot be done easily. Explain how the sample size was determined. Fifty-one is enough for inter-rater and test-retest reliability but not enough for a regression analysis. Make inclusion criteria of ADL and MMSE clearer. What are the compositions of 107 participants? Fifty-one older adults, 47 caregivers, and 5 VHS make only 103. 
4. Personal characteristics should include those related to a fall risk, in addition to demographic information. For example, falls in past one year, no falls or no vicarious falls so far, experiences in fall related injury and hospitalization, MMSE and ADL scores, readiness to make home safer, dizziness, balance problems etc. should be included. However, the sample size is too small for a multiple regression.
5. In Discussion, "...the group who were working in their own homes as housekeepers..." does not make sense. Revise this sentence. 
6. When we deal with human being for research, we use the word "participants" rather than "subjects". In Table 1, the statistical comparisons among these three groups are made; however, it is not the purpose of this study; therefore, they are not necessary. Descriptive statistics is sufficient. 
7. Overall impression of this study is that authors may be able to do more meaningful analyses using existing data for the personal characteristics part, although it has to be based on hypotheses derived from literature review. 

Is the work clearly and accurately presented and does it cite the current literature?

Partly

If applicable, is the statistical analysis and its interpretation appropriate?

Partly

Are all the source data underlying the results available to ensure full reproducibility?

Partly

Is the study design appropriate and is the work technically sound?

Partly

Are the conclusions drawn adequately supported by the results?

Yes

Are sufficient details of methods and analysis provided to allow replication by others?

Partly

Reviewer Expertise:

Fall prevention, smart house, e-health in gerontological population.

I confirm that I have read this submission and believe that I have an appropriate level of expertise to confirm that it is of an acceptable scientific standard, however I have significant reservations, as outlined above.

## References

[1] : Psychometrics of the Home Safety Self-Assessment Tool (HSSAT) to prevent falls in community-dwelling older adults. *Am J Occup Ther* .2014;68(6) : 10.5014/ajot.2014.010801 711-8 10.5014/ajot.2014.010801 25397766

# Response

**Published:** 2023-02-19

**Authors:** Charupa Lektip

**Affiliations:** Walailak University, Thailand

1. One of the most comprehensive studies about the reliability and validity of home hazards to prevent falls in older adults is missing. 

**Answer:** I will include that study in the discussion section of my research to discuss the similarities and differences of reliability and validity.

 2. This study is a methodological study not cross-sectional

**Answer:** This study is a prospective study

 3. A study replication cannot be done easily. Explain how the sample size was determined. Fifty-one is enough for inter-rater and test-retest reliability but not enough for a regression analysis. Make inclusion criteria of ADL and MMSE clearer. What are the compositions of 107 participants? Fifty-one older adults, 47 caregivers, and 5 VHS make only 103. 

**Answer:** - 51 elderly people were adequate for examining inter-rater reliability and test-retest reliability following the reference in the article" Rating the methodological quality in systematic reviews of studies on measurement properties: a scoring system for the COSMIN checklist" that recommends 50-99 subjects are Good sample size.

                   - I think a sample of 51 is sufficient for a regression analysis because correlation statistics will use 10-20 times the variable. And my main objective studies about the reliability and validity of home hazards to prevent falls in older adults.

                   - I exclude those who were completely dependent or disabled (ADL scores: 0-4) and those who received no formal education had MMSE-Thai scores of ≤14, when the elderly who received only upper secondary education had scores of ≤17, and when the elderly who continued their education received a score of ≤22.

                   - The compositions of 107  participants - Fifty-one older adults, 51 caregivers, and 5 VHV.

 4. Personal characteristics should include those related to fall risk, in addition to demographic information. For example, falls in the past year, no falls or no vicarious falls so far, experiences in fall-related injury and hospitalization, MMSE and ADL scores, readiness to make home safer, dizziness, balance problems, etc. should be included. However, the sample size is too small for multiple regression.

**Answer: **I think the variables you've suggested are very helpful in regression analysis. But with a small sample size, because the main objective of my work is to find reliability and validity, I only take personal characteristics variables that I show in the demographic table characteristics of study subjects.

 5. In Discussion, "...the group who were working in their own homes as housekeepers..." does not make sense. Revise this sentence. 

**Answer:** I revise it to " This research found that the group of people who acted as housekeepers in their own homes had a higher risk of falling, which may be related to the amount of clutter in the home.

 6. When we deal with a human being for research, we use the word "participants" rather than "subjects". In Table 1, the statistical comparisons among these three groups are made; however, it is not the purpose of this study; therefore, they are not necessary. Descriptive statistics are sufficient. 

**Answer:** -  I will use "participants" rather than "subjects". 

                    -  I will only use descriptive statistics to descript the three groups.

 7. Overall impression of this study is that authors may be able to do more meaningful analyses using existing data for the personal characteristics part, although it has to be based on hypotheses derived from the literature review. 

**Answer:** Thank you for your impression, I will add the literature review to support the personal characteristics associated with home hazards.
